# Examining the Outcomes of Project ECHO^®^ as an Interprofessional Community of Practice for Pediatric Feeding Clinicians

**DOI:** 10.1007/s00455-023-10603-z

**Published:** 2023-07-16

**Authors:** Madeline Raatz, Elizabeth C. Ward, Perrin Moss, Claire Reilly, Nadine Frederiksen, Corrine Dickinson, Sally Clarke, Kelly Beak, Jeanne Marshall

**Affiliations:** 1 Queensland Children’s Hospital, Children’s Health Queensland, PO Box 3474, South Brisbane, QLD 4101 Australia; 2 School of Health & Rehabilitation Sciences, The University of Queensland, Brisbane, QLD Australia; 3 Centre for Functioning and Health Research (CFAHR), Metro South Hospital and Health Service, Brisbane, QLD Australia

**Keywords:** Pediatric feeding disorder, Deglutition disorders, Training, Project ECHO, Telementoring, Multidisciplinary

## Abstract

Project ECHO^®^ is a virtual, interprofessional, cased-based peer-learning model. To date, no studies have explored ECHO as a model for pediatric feeding education. This study examined the outcomes of establishing a pediatric feeding ECHO network. Using a prospective, mixed-methods design, two cohorts of allied health professionals were recruited. Each cohort participated in eight, 90-min videoconference sessions incorporating a didactic presentation and clinical case presentation. The case was presented by a participant, with questions and recommendations provided by the ECHO network. Participants completed: (1) a learning needs analysis before the ECHO series, (2) a self-reported confidence questionnaire pre, post, and 3-month post, (3) a satisfaction questionnaire after each session, and (4) an overall satisfaction questionnaire post-ECHO series. Time spent by hospital allied health clinicians providing impromptu phone/email feeding support to external clinicians was recorded for 8 weeks prior to and 8 weeks during the ECHO series. Forty-seven participants were included in the study, attending an average of 5.8 sessions. Significant improvements in self-reported confidence were observed across the three time points (*p* < 0.01) with less experienced participants demonstrating greater improvements. Participants reported high satisfaction with ECHO, with 93% (40/43) wanting continued access to ECHO in future. The multidisciplinary format, interactivity, structure, and case-based nature of ECHO were considered beneficial. A 75% reduction in requests for support from clinicians in the same catchment area was noted during the ECHO series. Results demonstrated that Project ECHO is a viable model for pediatric feeding education for clinicians working in the field. Further research is needed to investigate the long-term effects and impacts on clinical care.

## Introduction

Pediatric feeding disorder (PFD) is defined as “impaired oral intake that is not age appropriate and is associated with medical, nutritional, feeding, skill and/ or psychosocial dysfunction” [1, p. 128]. This disorder can impact a range of clinical populations across childhood and can have detrimental impacts on health, nutrition, growth, development, and quality of life for children and their families [1–3]. The prevalence of PFD is increasing, due to improving survival rates of children with complex medical conditions and with the growing prevalence of neurodevelopmental disorders and chronic health conditions [4]. Multidisciplinary management is considered best practice due to the multifactorial nature of PFD [1, 5], and assessment and/or intervention most commonly involves practitioners from the disciplines of speech pathology, occupational therapy, dietetics, psychology, and medicine [5]. However, children’s access to PFD services can be negatively impacted by a range of factors including limited service availability and limited access to skilled providers [2, 6].

Unfortunately, many clinicians self-report low levels of confidence working with children with PFD [7–10]. This can result in PFD clinical expertise and experience remaining at certain facilities (e.g., tertiary hospitals, specialist feeding clinics), limiting the availability of services for families and/or adding additional travel burden [2, 6]. In addition, clinicians working in settings with expertise with PFD may be contacted for clinical support and advice, and/or to provide specialist consultations which may add to the burden of their own workload.

Although there are many theory-based and introductory pediatric feeding professional development (PD) opportunities, there has been a recognized need for intermediate to advanced level education, as well as practical, case-based PD events to support clinicians [7]. Recent research has emphasized the need for virtual training opportunities, to alleviate some of the barriers impacting training access and to increase the equity of training access for professionals from non-metropolitan areas [7].

A community of practice is a group of people who engage on a shared topic of interest as a means of sharing knowledge, learning, promoting professional expertise, and improving practice and performance [11, 12]. Communities of practice (CoP) can have varied membership (e.g., workplace specific single discipline membership vs. cross-sector multidisciplinary membership) and use varied methods of communication and interaction including in-person meetings, videoconferencing, and/or other virtual communication modalities (e.g., emails, online discussion board) [11]. Regardless of membership or communication method, most CoP aim to support learning and information/knowledge exchange and/or to improve practice [11]. Within the literature, outcomes of CoP have included (1) improved expertise, efficiency and knowledge, (2) increased access to networking, collaboration and resources, and (3) improved job satisfaction and retention [12, 13].

**Fig. 1:**
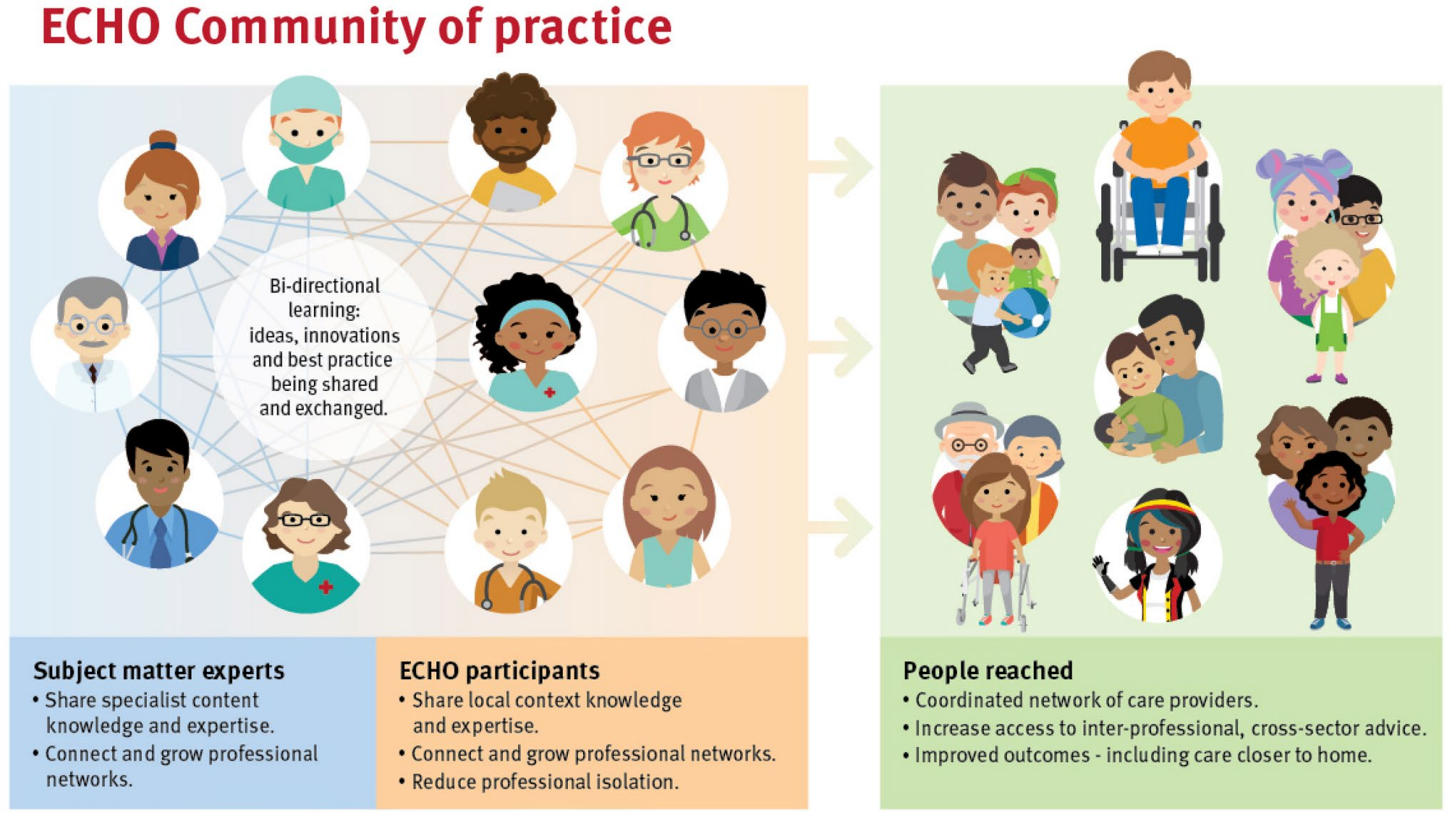
Project ECHO^®^ Community of Practice visualization (Copyright: Children’s Health Queensland Hospital and Health Service)

Project ECHO^®^ (Extension for Community Healthcare Outcomes) is a licensed and trademarked virtual knowledge-sharing model used to create communities of practice. It incorporates case-based learning strategies from medical education and theoretical frameworks including Social Cognitive Theory, Situated Learning Theory, and Community of Practice Theory [14, 15]. The ECHO model was initially developed as a platform for improving both service delivery and patient outcomes in treating Hepatitis C [16] and , however, has expanded to many areas of healthcare over time. Project ECHO is run virtually to reduce geographical, cross-sector, and professional access barriers [17–26]. It aims to support interprofessional education and capacity building through case-based learning strategies. Figure 1 provides a visual representation of the stakeholders involved in Project ECHO.

Given the success of ECHO in other areas of healthcare, this research aimed to examine the outcomes of establishing a pediatric feeding ECHO network (PedFeed ECHO). This particular network aimed to support clinicians who were working with children with PFD, and who were receiving limited local supervision, or had restricted access to peer networks. It was hypothesized that participants would report increased knowledge and confidence following their engagement with the PedFeed ECHO network. Secondary aims were to evaluate participant satisfaction with, and perceptions of, PedFeed ECHO and to examine the impact of PedFeed ECHO on requests for phone and/or email support from external clinicians seeking advice from a specialist, state-wide hospital-based feeding team.

## Methods

This research involved a prospective, mixed-methods design. Participants were invited to attend the PedFeed ECHO network with data collected before, during, and after the PedFeed ECHO series. The study was conducted with ethical approval from Children’s Health Queensland (LNR/21/QCHQ/75335) and all participants provided informed consent prior to participation.

### Participant Recruitment and Eligibility

Allied health professionals (AHPs) were invited to participate in the project through an expression of interest (EOI) disseminated via email through a variety of existing networks (e.g., special interest groups, professional associations). AHPs in this study included speech pathologists, occupational therapists, dietitians, and psychologists, working in the state of Queensland, Australia, which has an area of 715,309 square miles (1,859,642 square kilometers). The EOI included the participant information sheet and a survey that collected basic demographic information (e.g., location, profession, years’ PFD experience) to support assessment of project eligibility. Given that many of these existing networks had high representation of speech pathologists and/or health-based workers, the EOI was also emailed to 23 non-government organizations and private practices. These organizations and practices were identified through an internet search for providers advertising PFD services within Queensland to diversify the recruitment pool. Email recipients were also encouraged to forward the EOI to other relevant colleagues to increase potential sample size and diversity. To be eligible to participate in the research project, participants had to (1) be a speech pathologist, occupational therapist, dietitian, or psychologist, (2) provide services in Queensland, (3) have > 6 months experience providing services to children with PFD, and (4) provide services to children with PFD at least once per month. A sample target of 80 AHPs was set (40 × AHPs per group) based on recommended ECHO group size and anticipating 20% attrition over the course of the program. Clinicians with > 6 months experience who were actively seeing PFD patients were targeted to ensure that there would be adequate cases for presentation and to support robust peer discussion. Following review of the EOIs, participants were invited to participate in the project or notified that they were ineligible.

### ECHO Group Allocation

Participants were divided into two groups based on (1) availability and (2) maximum variation sampling targeting discipline, location, and workplace setting. The sessions for Group 1 were scheduled monthly (September 2021–April 2022) and Group 2 was scheduled fortnightly (November 2021–March 2022) as per participants’ availability and preferred session frequency. Both groups had a hiatus over the Christmas/New Year period (Group 1 = 6-week hiatus, Group 2 = 3-week hiatus). Once allocated to their group, participants were notified of session dates/times and asked to withdraw if they did not anticipate being able to attend at least 6/8 sessions.

### ECHO Sessions

Each group was offered eight 90-min sessions conducted via videoconference using the Zoom^®^ software platform. Each session had an interprofessional 4–5-person panel (i.e., subject matter experts as per Fig. 1) consisting of two speech pathologists, and a psychologist, dietitian, and/or occupational therapist. As per the ECHO model’s fidelity requirements, each session had both a facilitator and coordinator (both speech pathologists) and these professionals attended training in the coordination and facilitation of ECHO prior to series commencement. Fidelity of the program was maintained as per ECHO requirements, with minor adjustments made each session based on participant feedback. Consistent with other global ECHO networks, each session incorporated a brief didactic presentation on a PFD topic (e.g., food allergies, tube weaning, sensory processing) and a de-identified case presentation and discussion. Each case presentation was brought by a PedFeed ECHO participant from his/her own caseload and received recommendations from the PedFeed ECHO group and interprofessional panel. This enabled the case presenter to apply his/her new knowledge and group recommendations immediately following the session. Participants were emailed resources (e.g., slide handouts, summary of case presentation, and recommendations) after each session.

### Data Collection

Participants completed several questionnaires at various timepoints during the program as detailed below. Each of these questionnaires was distributed electronically via SurveyMonkey^®^ and is described below. Participants were allocated a participant number so all data were collected in a re-identifiable format.

- *Learning needs analysis (LNA)* The LNA was a 9-item questionnaire that aimed to investigate participants’ (1) learning objectives and priorities for the PedFeed ECHO series, (2) barriers to PFD service provision, (3) preferred learning modalities, and (4) session availability. This questionnaire was completed prior to the PedFeed ECHO series. Questions were either multiple choice (*n* = 6) or free-text responses (*n* = 3). The information gathered from this questionnaire was used to inform the content of the didactic presentations and the timing/frequency of the PedFeed ECHO series.
- *Self-reported confidence questionnaire* A 10-item questionnaire adapted from Furlan et al. [27] was used to investigate participants’ self-reported confidence assessing and managing clients with PFD. This questionnaire was completed prior to, immediately post-, and 3 months post-ECHO series. Most questions (*n* = 9) were scored on a 5-point Likert Scale (where 1 = strongly disagree and 5 = strongly agree) with one visual analog scale (0–100, not at all confident to very confident). An additional Likert question (*n* = 11) was added to the post-ECHO version of the questionnaire (“Do you feel that PedFeed ECHO has contributed to changes in your confidence?”).
- *Post-session satisfaction questionnaire* A post-session satisfaction questionnaire was sent after each PedFeed ECHO session. It included 6 questions (3 binary choice, one 5-point Likert scale, and 2 free-text responses) to investigate attendance (including reason for non-attendance), if the session met participant learning needs, key session learnings, general feedback/suggested modifications, and an opportunity to volunteer for the next case presentation. Information from this questionnaire was used to modify/improve future sessions.
- *Overall satisfaction questionnaire* The overall satisfaction questionnaire was modified from Arora et al. [28] and Tantillo et al. [29] and was completed after the PedFeed ECHO series. It included 14 questions (11 5-point Likert scale and 3 yes/no) to investigate participants’ satisfaction with and outcomes from participation in the PedFeed ECHO series (e.g., “involvement in the PedFeed ECHO network was a worthwhile experience”). In addition, participants were also asked to provide comment on what aspect/s of the PedFeed ECHO network they felt worked well, change/s they would recommend for future PedFeed ECHO cohorts, and how they felt PedFeed ECHO was different from other PD they had attended.

Participants who attended ≤ 2 sessions within the 8-session series were sent a separate 5-item questionnaire to understand f the actor/s that contributed to non-attendance. This included both forced choice (*n* = 2) and open-ended (*n* = 3) response options.

### Time Capture

A purpose-built time-capture questionnaire was completed by clinicians at the tertiary hospital to record the time spent providing advice/support to external clinicians regarding patients with pediatric feeding needs (not related to clinical handover). Clinicians providing PFD services at the tertiary hospital were identified, and asked to complete the questionnaire at times when they received support requests, with informed consent incorporated into the questionnaire landing page. This support was provided to clinicians in the same catchment area, but not necessarily provided to clinicians who were participating in the PedFeed ECHO series. The questionnaire contained 12 questions incorporating both forced choice (*n* = 10), numerical (*n* = 1) and open-ended (*n* = 1) questions. Respondents were asked about the individual seeking support (e.g., profession, sector) and the support request (e.g., outcome of the phone call/email, time taken to respond). Data were collected over an 8-week period prior to the launch of PedFeed ECHO and for 8 week when the PedFeed ECHO series was being run.

### Data Analysis

The Accessibility Remoteness Index of Australia (ARIA) [30] was used to categorize respondents’ geographical location as metropolitan or non-metropolitan based on their reported postcode [30, 31]. Frequency counts and percentage response were used to analyze response frequency. All statistical analyses were conducted using IBM SPSS Statistics version 27. Differences in demographic characteristics between the two groups were compared using Chi-square tests. Self-reported confidence ratings were compared across the three time points (pre, immediately post, and 3-month post), with one-way repeated measures analysis of variance (ANOVA) used for continuous data and Friedman tests used for ordinal data. Post hoc analysis was completed using a paired t-test for continuous data and the Wilcoxon signed rank test for ordinal data, with a more conservative *p* value of < 0.01 applied. Overall satisfaction between the two groups was compared using the Mann–Whitney *U* test. Sub-analyses were completed using a repeated measures ANOVA test considering differences in confidence and satisfaction measures according to geographic classification, profession, employment setting, frequency of service provision, years’ PFD experience, and PFD caseload proportion.

Plain content analysis [32] was used to explore the open responses from the three free-text questions in the overall satisfaction questionnaire. Due to the nature of these responses, content analysis (as opposed to thematic analysis) was considered most appropriate, as it allows the key meanings and patterns across qualitative data to be reported in categories/subcategories, using frequency counts. For the analysis, two authors (J.M and M.R) individually read approximately one third of the free-text responses to generate the initial codes and potential categories. These two authors then met together to discuss these and reach consensus on the final set of codes and categories. The first author (M.R) then coded and categorized all remaining comments and counted code frequency.

## Results

**Fig. 2:**
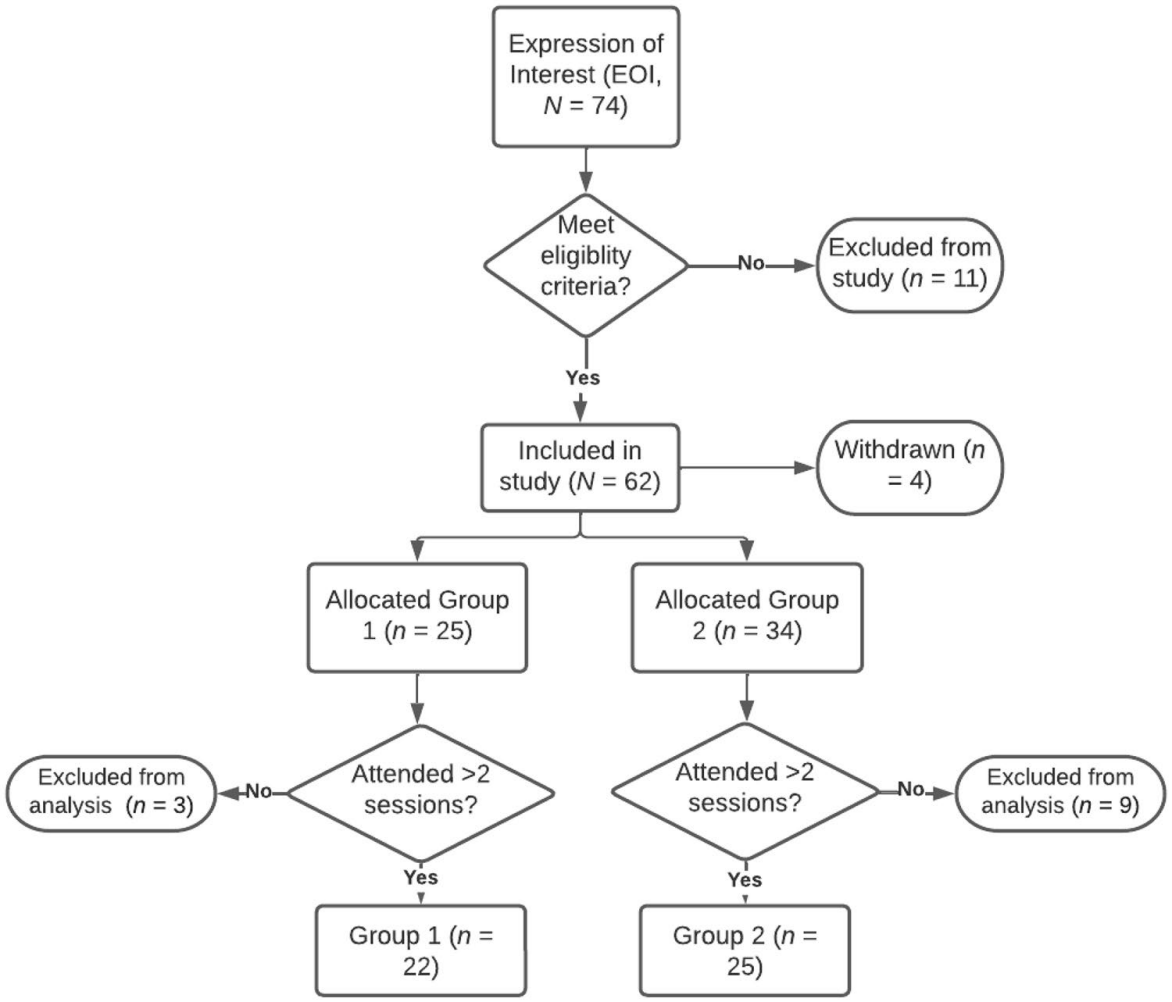
Overview of participant recruitment, allocation, and retention in PedFeed ECHO. Created in Lucidchart (www.lucidchart.com)

A total of 74 clinicians expressed interest in participating in the PedFeed ECHO network with 47 included in the study. As outlined in Fig. 2, 11 participants were excluded as they did not meet eligibility criteria, including being located outside of Queensland (*n* = 4), having < 6 months experience with PFD (*n* = 4), and not being an AHP (*n* = 1). Two participants did not return their enrollment forms before the commencement of the groups, and so were also excluded. Overall, 12 participants were excluded from data analysis as they attended ≤ 2 sessions. Of the participants who attended ≤ 2 PedFeed ECHO sessions, 50% (*n* = 6) were speech pathologists and 50% (*n* = 6) were dietitians, with most (*n* = 7, 58%) residing in non-metropolitan locations. Four of these non-attenders (33%) completed the non-attendance questionnaire and reported reason/s that they were unable to attend. Reported reason/s (some participants reported > 1) included session time/day unsuitable (*n* = 2), change of role (*n* = 2), and inability to prioritize with other work/clinical demands (*n* = 2). All four non-attenders who provided feedback (100%) reported accessing the resources distributed via email after each session and found these useful.

### Participant Demographics

Participant demographics for the final set of participants (*n* = 47) are outlined in Table 1. The majority of participants were speech pathologists (*n* = 32, 68%), had been providing pediatric feeding care for < 5 years (*n* = 30, 63%) and provided pediatric feeding care at least once per fortnight (*n* = 40, 87%). There was no statistically significant difference in demographic variables between Group 1 and Group 2. Almost all of the cohort (*n* = 44, 94%) reported that they had previously attended pediatric feeding PD event/s (*n* = 3, 6% nil previous pediatric feeding PD, *n* = 1, 2% no response). When asked about their preferred learning methods respondents indicated preference for in-person teaching (*n* = 40, 83%) and webinars (*n* = 39, 81%) over other types of learning (online learning modules *n* = 35, 73%; podcast *n* = 26, 54%; teleconference *n* = 33, 69%).

**Table 1:** Participant demographic information (*N* = 47)

Demographics	Whole group *n* (%)	Sub-analysis by group *n*(%)		*χ*^2^	*p* value
		Group 1 (n = 22)	Group 2 (n = 25)		
Profession				1.25	0.74
Dietitian	9 (19)	4 (18)	5 (20)		
Occupational therapist	5 (10)	2 (9)	3 (12)		
Psychologist	1 (2)	1 (5)	0 (0)		
Speech pathologist	32 (68)	15 (68)	17 (68)		
Workplace location				2.69	0.10
Major city	25 (53)	15 (68)	10 (40)		
Regional	22 (47)	7 (32)	15 (60)		
Employment setting				4.17	0.38
Community health	8 (17)	6 (27)	2 (8)		
Disability services	3 (6)	2 (9)	1 (4)		
Education	4 (9)	2 (9)	2 (8)		
Hospital	21 (45)	8 (36)	13 (52)		
Private practice	11 (23)	5 (23)	7 (28)		
Years’ experience providing PFD services				6.52	0.16
6 months–2 years	19 (40)	6 (27)	13 (52)		
3–5 years	11 (23)	4 (18)	7 (28)		
6–9 years	9 (19)	6 (27)	3 (12)		
10–20 years	7 (15)	5 (23)	2 (8)		
> 20 years	1 (2)	1 (5)	0 (0)		
Age group/s PFD services provided to					
Preterm infants	20 (43)	9 (41)	11 (44)	0.00	1.00
Neonates (0–3 months)	29 (62)	13 (59)	16 (66)	0.00	0.96
Infants (4–11 months)	37 (79)	18 (82)	19 (76)	0.17	0.90
Toddlers (1–2 years)	44 (94)	22 (100)	23 (92)	0.00	1.00
Pre-schoolers (3–5 years)	46 (98)	22 (100)	24 (96)	0.00	1.00
School aged (5–12 years)	37 (79)	17 (77)	20 (80)	0.00	1.00
Adolescents (13–18 years)	26 (55)	10 (45)	16 (64)	0.96	0.33
Frequency of PFD service provision				1.94	0.59
Frequently (≥ 1 per week)	32 (68)	15 (68)	17 (68)		
Regularly (≥ 1 per fortnight)	9 (19)	3 (14)	6 (24)		
Semi-regularly (≥ 1 per month)	2 (4)	1 (5)	1 (4)		
Irregularly (≥ 1 per 3-months)	4 (9)	3 (14)	1 (4)		
Percentage caseload PFD				1.99	0.85
< 10%	3 (6)	1 (5)	2 (8)		
10–25%	18 (38)	7 (32)	11 (44)		
26–50%	10 (21)	4 (18)	6 (24)		
51–75%	5 (11)	3 (14)	2 (8)		
76–90%	2 (4)	1 (5)	1 (4)		
> 90%	8 (17)	5 (23)	3 (12)		
No response	1 (2)	1 (5)	0 (0)		

*PFD* Pediatric feeding disorder; preterm infants refer to any infants born < 37 weeks gestational age; neonates in this study are full-term infants born ≥ 37 weeks of gestational age who are between 0 and 3 months of age (i.e., not preterm infants)

### Attendance and session satisfaction

Participants attended an average of 5.8 sessions within the PedFeed ECHO series (standard deviation [SD] 1.5; Group 1 average 6 sessions, SD 1.5; Group 2 average 5.7, SD 1.6). Group 1 (*n* = 22) had an average of 16.4 attendees per session (SD 4.4) and Group 2 (*n* = 25) had an average of 17 attendees (SD 3.8). There were 215 post-session satisfaction questionnaires completed by participants who attended sessions. Most participants agreed that each PedFeed ECHO session was useful for their clinical practice (*n* = 110, 51% strongly agree, *n* = 88, 41% agree, *n* = 17, 8% neutral, *n* = 0 disagree or strongly disagree). There were 63 post-session questionnaires completed by participants who did not attend a session. Reasons for non-attendance were reported to include annual leave (*n* = 17, 27%), clinical conflict (e.g., urgent inpatient *n* = 13, 21%), sick/carers leave (*n* = 12, 19%), scheduling conflict (*n* = 12, 19%), emergent leave (e.g., natural disaster *n* = 3, 6%), COVID-19 redeployment (*n* = 3, 6%), and forgot (*n* = 1, 2%).

### Self-reported confidence

Participants’ overall self-reported confidence increased over the course of the PedFeed ECHO series (pre- mean 61.3, SD 22.1; post-mean 75.9, SD 14.1; 3-month post-mean 77.1, SD 14.7). Statistical analysis identified a significant change in ratings across the three timepoints (Wilk’s Lambda = 0.49, F(2, 35) = 18.15, *p* < 0.01, effect size = 0.51). Post hoc analyses indicated a significant pre–post (*p* < 0.01)- improvement; however, no further significant change (*p* = 0.27) between the immediately post- and 3-month post-timepoints indicating maintenance of the post-PedFeed ECHO confidence level. When considering demographic characteristics, there was no significant difference in overall self-reported confidence according to geographical location (*p* = 0.89), profession (*p* = 0.58), employment setting (*p* = 0.20), frequency of service provision (*p* = 0.20), or proportion of PFD caseload (*p* = 0.50). However, there was a significant difference in post-PedFeed ECHO confidence when considering number of years’ experience working with children with PFD. Clinicians with less experience (6 months – 2 years) reported significantly greater improvement in self-reported confidence in comparison to participants with > 2 years’ experience (*p* = 0.002).

Average participant ratings on the self-reported confidence questionnaire are outlined in Table 2, with Friedman tests indicating significant differences in confidence across the three time points. Post hoc analysis indicated that the differences were significant between the pre- and post-PedFeed ECHO time points (i.e., pre–post and pre–3-month post; *p* < 0.01) but not between the two post-time points (i.e., post and 3-month post; *p* > 0.05) for all questions except “confidence seeking additional evidence” (*p* =  < 0.01 pre–post, *p* = 0.04 pre–3-month post). When participants were asked if they believed that the PedFeed ECHO series had contributed to their changes in confidence, most strongly agreed (*n* = 21, 57%) or agreed (*n* = 11, 28%) (neutral *n* = 3, 8%; no response *n* = 2, 5%).

**Table 2:** Self-reported confidence (*N* = 37)

Question (I am confident in my ability to…)	Median score			*χ*^2^	*p*
	Pre	Post	3-mth post		
Identify patients who need care for PFD	4.3	4.7	4.6	19.11	< 0.01*
Assess patients with PFD	3.8	4.2	4.2	14.49	< 0.01*
Provide treatment for patients with PFD	3.5	4.1	4	20.69	< 0.01*
Seek further evidence to support my patients with PFD	4.1	4.5	4.4	8.85	0.01*
Be identified as competent within my clinic and in my locality for PFD	3.6	4.3	4.1	21.00	< 0.01*
Work with families to support children with PFD	4.0	4.3	4.3	8.99	0.01*
Identify patients who may need referral to other disciplines	4.2	4.6	4.6	11.86	< 0.01*
Identify patients who may need referral to a tertiary center	3.9	4.6	4.5	21.07	< 0.01*
Communicate my recommendations with other staff in my facility who may not be as experienced with PFD	3.8	4.3	4.3	20.60	< 0.01*

1 = Strongly disagree, 3 = Neutral, 5 = Strongly agree

*PFD *pediatric feeding disorder

*p ≤ 0.05

### Overall satisfaction with PedFeed ECHO

Participants reported high satisfaction with the PedFeed ECHO series, with questionnaire responses detailed in Table 3. The satisfaction outcomes were positive across both groups; however, Group 2 reported significantly higher satisfaction with improvements to care and reduced feelings of professional isolation as compared to Group 1. Almost all of the cohort (*n* = 40/43, 93%) indicated that they wanted to continue accessing PedFeed ECHO and the majority (*n* = 32, 74%) reported preferring to access PedFeed ECHO over traditional lecture-style workshops in future.

**Table 3:** Overall satisfaction questionnaire (*N* = 42)

Question	Whole group	Group 1 (*n* = 19)	Group 2 (*n* = 24)	Mann–Whitney *U*	Effect size	*p*
Involvement in PedFeed ECHO was worthwhile	4.5	4.3	4.5	181	− 0.15	0.32
I would recommend PedFeed ECHO to my colleagues	4.6	4.6	4.6	227	− 0.004	0.98
Participation in PedFeed ECHO has enhanced my professional satisfaction	4.3	4.3	4.4	218	− 0.4	0.80
PedFeed ECHO has enabled rapid learning and best practice dissemination	4.3	4.3	4.3	223	− 0.02	0.99
PedFeed ECHO has improved the safety of my pediatric feeding care	3.8	3.7	3.9	194	− 0.14	0.37
The quality of care I provide has improved as a result of PedFeed ECHO	4	3.7	4.3	134	− 0.38	0.01*
The goals and objectives I had upon becoming involved in PedFeed ECHO have been met	4.1	3.9	4.3	181	− 0.19	0.21
PedFeed ECHO has diminished my feelings of professional isolation	4.1	3.8	4.3	152	0.34	0.04*
I have developed new relationships with other providers that are of benefit to my service	3.4	3.2	3.6	179	− 0.2	0.21
PedFeed ECHO has expanded access to evidence-based care for pediatric feeding patients in our service	4.2	4.2	4.2	217	− 0.5	0.77
PedFeed ECHO has reduced variations in care across the state	3.6	3.5	3.7	192	− 0.15	0.33

1 = strongly disagree, 3 = neutral, 5 = strongly agree

*p < 0.05

Content analysis of the open-ended questions included in the overall satisfaction survey is reported in Tables 4 and 5. All participants provided responses regarding what worked well and the difference between PedFeed ECHO and other professional development opportunities, and this information is summarized across five categories in Table 4. Overall, a number of respondents reported perceiving that real and current cases were valuable, ECHO structure was good, the interactivity and opportunity for questions, discussion and participation was valuable and that the multidisciplinary format was beneficial. Suggestions for improvements for future PedFeed ECHO cohorts were analyzed separately and are outlined in Table 5. Only 23 participants provided suggestions for program improvement. From their responses, there were 7 categories identified. Due to the low response rate, most codes came from only one comment, with the exception of wanting to exchange contact information, wanting a smaller group size and wanting more time for didactic teaching.

**Table 4:** Perceptions of positive aspects of PedFeed ECHO and/or PedFeed ECHO in contrast with other professional development

Category	Code	Number of comments	Example comment/s
Flexible, practical, and responsive learning opportunity	Real and current cases were valuable	12	“The opportunity to hear a case presentation and listen to participant and panel members' feedback was wonderful. Having case-based discussion where practical advice could be offered and panel members could show how they would apply feeding knowledge/skill was much more meaningful”
	Content was flexible, responsive and tailored to group needs	11	“I feel that this was more clinically relevant than other PDs as we spoke about things like family circumstances etc. that aren't discussed in PDs” “It was personalised to the needs of the group attending”
	Sharing of resources was beneficial	3	“Sharing of resources from all professionals present. This allowed ideas that have been found to be useful in different settings to be shared and from different disciplines…”
	Context-specific learning was valuable	2	“I think the ECHO group was more relevant to clinicians working in multi-disciplinary feeding clinics within Queensland”
	Multiple sessions over a longer period were helpful	2	“I liked the short sessions spaced over a longer period of time. It allows for greater absorption of material and deeper reflection and opportunity for implementation of ideas”
	Opportunity for multidisciplinary case presentations was helpful	1	“Allowing 2 clinicians to present a joint case worked well. I feel this option may encourage more people to present”
ECHO format and structure	Enjoyed interactivity and opportunity for questions, discussion and participation	19	“I like that it was more case-based and interactive—this made me think more about applying knowledge and using clinical reasoning rather than just learning content” “It was great to be able to seek advice, feedback, and to have a dialogue with the other ECHO network members and panel”
	Structure was good	11	“I liked the combination of presentation and case discussion”
	Multidisciplinary format was beneficial	8	“Having access to experienced professionals from the relevant allied health team at the one-time enhanced the information shared and the quality and depth of discussion about each case”
	Opportunity to learn from experienced panel members was helpful	4	“Ongoing access to presenters who specialise in feeding helped me feel more connected and supported” “Having case-based discussion where practical advice could be offered and panel members could show how they would apply feeding knowledge/skill was much more meaningful”
	Live case summary was helpful	3	“Note taking by the panel member during the case presentation was useful”
Community of practice	Peer learning	4	“Enjoyed having the same group of people to discuss and learn from” “I liked the networking and peer to peer support style of this PD”
	Sense of community	3	“ECHO allowed for a greater sense of comradery -'We are all moving in this space together and having similar experiences'”
	Opportunity to learn from others in different settings	2	“I loved that participants were working across acute and community-based settings…”
Group dynamics influenced psychological safety	Panel supported safety and inclusion	4	“Fairly early on there was a sense of 'safe space' where people could share their challenges” “Panel did a wonderful job making people feel safe, valued, included”
	Group size supported participation	1	“I enjoyed that it was a smaller group and you didn't feel embarrassed to make suggestions or ask questions”
Virtual medium	Accessible	6	“ECHO was great because it was accessible” “On-line attendance…allow[ed] access to multiple professionals from different settings”
	Participants having cameras on supported sense of camaraderie	2	“I think ensuring everyone keeps their cameras on is a good idea to encourage participation”

**Table 5:** Improvements/recommendations for future PedFeed ECHO cohorts

Category	Code	Number of comments	Example comment/s
Flexible, practical, and responsive learning opportunity	Wanted more flexibility with session timing	1	“Fortnightly sessions were lovely but I can see it becoming a little difficult to commit when there are only a couple of times available”
ECHO format and structure	ECHO cohorts focused on specific pediatric feeding themes would be helpful	1	“Ideally, different ECHOs for different aspects of care in paediatric dysphagia”
	Wanted to receive case ahead of session	1	“For future cohorts, it would be good to consider providing the outline of the case to all participants as few days in advance to allow time to access relevant resources that might be useful to share and consider the case in more depth”
	Wanted post-ECHO series debrief session	1	“I would have benefited from a post-ECHO series session to discuss with my cohort themes that may have arisen/learnings and how I am planning to make changes to my practice as a result of participating”
	Wanted addition of further audio-visual information	1	“I know there may be privacy issues, but if there were videos or photos of the cases we were discussing, it would provide even further insight”
	Wanted more structure for case presentation and discussion	1	“More consistency around the case discussion and how/what information is given”
Community of practice	Wanted to exchange contact information	4	“An opportunity to exchange contact details might be useful”
Group dynamics influenced psychological safety	Wanted smaller group size	3	“I would prefer a smaller group size, e.g. 8–10 participants”
	Not all peers participated equally	1	“Would be great to have more…managing [of] people [who] potentially always answer”
	Wanted more opportunities to establish safety in community	1	“I wonder about activities that could occur prior to the series commencing to support a sense of camaraderie and "safety" amongst the participants”
	Didn’t feel confident to contribute	1	“It was challenging for me as an introverted person to feel confident offering feedback to other participants, [however] my confidence in this grew toward the end of the series”
Virtual medium	Difficulty forming strong relationships in a virtual medium	1	“It is challenging to form strong relationships with people over zoom”
	Virtual medium intimidating to participate	1	“I personally find speaking up and contributing over telehealth a little intimidating and am generally more confident face to face”
Time allocation	Wanted shorter sessions	1	“Shorter length of sessions”
	Wanted more time for session overall	1	“The pausing was helpful, however often I felt as though I walked away with questions because I had time to "let it sink in"
	Too much time spent on some aspects of sessions	1	“The clarifying questions and the recommendations could become lengthy”
	Wanted more time to hear from panel	1	“I would have enjoyed less time with the peers speaking and more time from the panel summarizing and making further recommendations”
	Wanted more time for didactic teaching	2	“Could have spent more time on this [didactic]”
	Wanted more time at next session to discuss reflections	1	“More opportunity to ask follow up questions, post reflection and then have some clarification at the following ECHO would be great for learners who are also like this”
ECHO did not meet everyone’s needs	Participants needed foundational feeding knowledge to contribute	1	“Personally, I am lacking in some of the foundational skills from PDs before I am ready to be an active contributor in complex case discussions”
	Not deep enough learning for some participants	2	“Perhaps only a surface level of knowledge was attainted, rather than in depth and robust understanding”
	Difficulty applying learning across all contexts	1	“The content was quite health-heavy. Great for my learning and awareness, but also quite tricky to follow at times or apply to community contexts”

### Time

In the 8 weeks prior to commencement of the PedFeed ECHO series, 27 requests for support were received by the specialist PFD service (*n* = 18, 67% via phone; *n* = 9, 33% via email). Overall, 25 requests were fielded by speech pathology staff (93%), and two by dietetics staff (7%). A total of 498 min (8.3 h) was spent responding to requests, with the average response taking 18.4 min (range 5–45 min). Most of these requests (*n* = 21, 78%) were for clinical advice/support (*n* = 6, 22% regarding potential referrals) and the outcome of almost all (*n* = 25, 93%) support was the provision of clinical advice (*n* = 2, 7% resulted in referral to the service). While most requests were received from professionals working within the health sector (*n* = 19, 70%), a small number (*n* = 8, 30%) were received from professionals in other sectors (e.g., private practice, disability, community health). Speech pathologists (*n* = 24, 89%) were the most frequent professionals contacting to request support (dietitian *n* = 2, 7%, nurse *n* = 1, 4%). Most of the requests (*n* = 16, 59%) were not clinically urgent.

During the 8 weeks when the PedFeed ECHO series was run, there was a 75% reduction in requests for support, with 7 requests received (*n* = 4, 57% via phone; *n* = 3, 43% via email). All requests for support were fielded by speech pathology team members (n = 7, 100%). A total of 125 min (2.1 h) was spent providing support, with the average response taking 17.9 min (range 5 – 30 min). Most of these requests (*n* = 5, 71%) were for clinical advice/support (*n* = 2, 29% regarding potential referrals) and the outcome of all requests (100%) was the provision of clinical advice. The majority of requests were received from professionals working outside the health sector (*n* = 4, 57%). Speech pathologists (*n* = 6, 86%) were the most frequent professionals contacting for support (occupational therapist *n* = 1, 14%). Most (*n* = 5, 71%) of the requests were not clinically urgent.

## Discussion

This study demonstrated that Project ECHO is a viable model for pediatric feeding education. Overall, participants reported increased confidence following the PedFeed ECHO series, high satisfaction with PedFeed ECHO, and the PedFeed ECHO period that was observed to coincide with a reduction in external support requests by the hospital service. Additionally, most respondents requested to continue accessing the PedFeed ECHO network in future and indicated a preference for the community of practice ECHO model over traditional lecture-style teaching. Self-reported increases in knowledge and confidence after participation in the PedFeed ECHO series is consistent with previous research [18, 24, 25, 27, 28, 33, 34]. Notably, these improvements in self-reported confidence were maintained, but did not continue to increase 3-months after completion of the PedFeed ECHO series indicating that participation in the PedFeed ECHO continued to have impact after cessation of the sessions. Although there are known limitations to self-reported confidence ratings [35, 36], this finding is important given that previous research has demonstrated that knowledge and confidence impact clinician’s anxiousness to provide pediatric feeding care [7]. Of note, clinicians with less experience reported the greatest increase in self-reported confidence, likely attributable to their lower levels of confidence prior to participation. Interestingly, these improvements in confidence were also observed to coincide with reduced requests for clinical support; potentially indicating that clinicians felt more confident in their clinical decision-making and/or that the opportunity for case-based learning reduced the need for individual case-based support and advice.

Participants reported being highly satisfied with PedFeed ECHO as a learning modality, which is similar to previous studies using this model [21, 24, 25, 28]. In particular, participants reported highly valuing the interactive nature of PedFeed ECHO and the combination of tailored didactic presentations and case-based learning. While these components have been identified as benefits in previous ECHO research [17–19, 24], literature within the medical and nursing fields recognizes that interactive teaching techniques and case-based learning are more effective than traditional didactic lectures at improving individual performance and patient outcomes [37, 38]. Indeed, within free-text comments participants reported that the use of current cases, as well as the opportunity to network and learn from others in different workplaces and from different professions was highly beneficial to their learning. Opportunities for networking, collaboration and varied membership are one of the key aims of the development of communities and practice, and are a commonly recognized benefit/outcome [12, 13] Indeed, within the literature both interprofessional education and peer learning have been demonstrated to lead to positive changes in attitude, collaborative knowledge and skill [17, 18, 21, 24, 39]. However, some participants within this study appeared to value the input from the PedFeed ECHO panel more highly than that of their peers. This may reflect limited insight into the expertise and potential benefits of peer-to-peer support; however, future research could aim to better understand these comments and perceptions.

Many participants reported that being able to participate in PedFeed ECHO remotely supported accessibility. The importance of providing clinicians with flexible and virtual pediatric feeding training options has been highlighted in previous research [7], and has been recognized as a benefit of previous ECHO studies [17, 18]. As ECHO is conducted virtually, it is possible to conduct sessions over an extended period of time. Previous research has demonstrated that PD activities conducted over longer periods of time and with multiple exposures (i.e., not an isolated one-day training activity) are most effective at improving performance and patient health outcomes [38]. Interestingly, while a small number of participants reported that multiple sessions were a benefit of the PedFeed ECHO model, the fortnightly cohort demonstrated significantly greater improvements on self-reported ratings of improvements in clinical care and reduction in feelings of professional isolation than the monthly cohort. It is hypothesized that the monthly gap between sessions for Group 1 was too long, resulting in reduced establishment of a community of practice and subsequently less impact on clinical care.

It is important to recognize that while PedFeed ECHO was positively received by the majority of participants, it did not meet everyone’s needs and a number of suggestions were provided to improve future PedFeed ECHO cohorts. In particular, several participant comments across both the fortnightly and monthly cohorts reflected on the establishment of psychological safety within the group, with comments centered around preferring a smaller group size or having more opportunity to build confidence and safety within the peer-group. There is also potential that some participants found this harder to achieve via the virtual medium used within PedFeed ECHO, with less opportunities for informal interaction and rapport building. The implementation of themed PedFeed ECHOs (e.g., acute pediatric feeding, tube weaning) as suggested by some participants in this study and within previous research [25] may further help to improve group dynamics, time allocation and deeper learning by further refining and narrowing session content and group discussion. Finally, there is limited information available regarding why a subsection of participants did not attend the PedFeed ECHO series or only attended 1–2 sessions. While the few respondents who completed the non-attendance questionnaire reported their reason/s as being due to change in role or difficulty with scheduling, it is possible that the other participants did not see it as valuable or as meeting their learning needs.

### Limitations

It is acknowledged that this study has a number of limitations. Firstly, the majority of participants were speech pathologists, with lower representation of other health professionals, although this is commensurate with the health professionals most frequently providing pediatric feeding services in Australia. Future research should aim to engage a wider representation of multidisciplinary professionals. Additionally, this study only examined the short-term impact (3 months) of engagement in the PedFeed ECHO network and did not use any direct measures of knowledge, professional practice/skill, or patient outcomes. Future research could incorporate these types of measures as well as a more robust research design such as a randomized controlled trial with a larger sample over a longer period to fully evaluate the effectiveness of this education model. Finally, it is acknowledged that the measure used to capture change in support requests was relatively crude. Although the staff providing support were from the same catchment as the participants accessing the PedFeed ECHO series, it was not clear as to whether those seeking support were also accessing PedFeed ECHO, and so attributing the change in support requests directly to the influence of PedFeed ECHO may be tenuous. The registration of such requests was also dependent on staff at our facility, who may have been more eager at the commencement of the project. Future research could investigate the impact of Project ECHO on support requests over longer periods of time and complete a robust economic analysis to compare the costs associated with ECHO versus standard practice.

## Conclusion

Project ECHO is a feasible model for pediatric feeding education, with participants reporting high satisfaction and improved self-reported confidence after engaging with this learning modality. The interactive, case-based nature of PedFeed ECHO meets the need for practical, remote training opportunities and the training was observed to result in reduced requests for clinical support. Future research should investigate the long-term impacts of participation in PedFeed ECHO, as well as the direct impact on clinical care.

## Data Availability

The datasets generated during and/or analyzed during the current study are available from the corresponding author on reasonable request.

## References

[1] Goday P, Huh SY, Silverman AH (2019). Pediatric feeding disorder—consensus definition and conceptual framework. J Pediatr Gastroenterol Nutr.

[2] Estrem HH, Thoyre SM, Knafl KA (2018). “It’s a long-term process”: description of daily family life when a child has a feeding disorder. J Pediatr Health Care.

[3] Taylor T, Taylor SA (2021). Let’s not wait and see: the substantial risks of paediatric feeding problems. Int J Child Adolesc Health.

[4] Kovacic K, Rein LE, Szabo A (2021). Pediatric feeding disorder: a nationwide prevalence study. J Pediatr.

[5] Sharp WG, Volkert VM, Scahill L (2017). A systematic review and meta-analysis of intensive multidisciplinary intervention for pediatric feeding disorders: how standard is the standard of care?. J Pediatr.

[6] Raatz M, Ward EC, Marshall J (2021). “It takes a whole day, even though it’s a one-hour appointment!” Factors impacting access to pediatric feeding services. Dysphagia.

[7] Raatz M, Marshall J, Ward EC (2023). Understanding training needs in pediatric feeding for allied health professionals: an Australian perspective. Am J Speech Lang Pathol.

[8] Ashton L, May J, Brook T, Clarke K. Multidisciplinary therapy services for children with feeding disorders in country South Australia. 2013. https://www.ruralhealth.org.au/12nrhc/wp-content/uploads/2013/06/Ashton-Larissa_May-Jodie_ppr.pdf. Accessed 16 Mar 2023.

[9] Zimmerman E. Pediatric dysphagia: a rise in preterm infants and a need for more formal training for speech-language pathologists. Int J Gynecol Obstet Neonatal Care 2016; 3:15–20. 10.15379/2408-9761.2016.03.01.03

[10] Marshall J, Hill RJ, Dodrill P (2013). A survey of practice for clinicians working with children with autism spectrum disorders and feeding difficulties. Int J Speech Lang Pathol.

[11] Ranmuthugala G, Plumb JJ, Cunningham FC (2011). How and why are communities of practice established in the healthcare sector? A systematic review of the literature. BMC Health Serv Res.

[12] Seibert S (2015). The meaning of a healthcare community of practice. Nurs Forum (Auckl).

[13] Bassi S, Polifroni EC (2005). Learning communities: the link to recruitment and retention. J Nurses in Staff Dev.

[14] Socolovsky C, Masi C, Hamlish T (2013). Evaluating the role of key learning theories in echo: a telehealth educational program for primary care providers. Prog Community Health Partnersh.

[15] Wenger E, McDermott R, Snyder W (2002). Cultivating communities of practice: a guide to managing knowledge.

[16] Arora S, Kalishman S, Dion D (2011). Partnering urban academic medical centers and rural primary care clinicians to provide complex chronic disease care. Health Aff.

[17] Arora S, Mate KS, Jones JL (2020). Enhancing collaborative learning for quality improvement: evidence from the improving clinical flow project, a breakthrough series collaborative with project echo. Jt Comm J Qual and Patient Saf.

[18] de Witt JB, Brazil K, Passmore P (2018). Evaluation of the impact of telementoring using ECHO© technology on healthcare professionals’ knowledge and self-efficacy in assessing and managing pain for people with advanced dementia nearing the end of life. BMC Health Serv Res.

[19] Gleason LJ, Beiting KJ, Walker J (2020). Using telementoring to share best practices on COVID-19 in post-acute and long-term care facilities. J Am Geriatr Soc.

[20] Joshi S, Gali K, Radecki L (2020). Integrating quality improvement into the ECHO model to improve care for children and youth with epilepsy. Epilepsia.

[21] Katzman JG, Gygi K, Swift R (2020). How hands-on pain skills intensive trainings complement echo pain and opioid management programs: a program evaluation with the Indian health service. Pain Med.

[22] Katzman JG, Herring D, Schramm P (2021). Climate change and human health echo: global telementoring for health professionals. J Med Educ Curric Dev.

[23] Lewiecki EM, Boyle JF, Arora S (2017). Telementoring: A novel approach to reducing the osteoporosis treatment gap. Osteoporos Int.

[24] McPhillips AM, Schultz RJ, Nasuta M, Shafer PO (2021). ECHO telementoring applied to managing students with seizures: the benefits for school nurses. NASN Sch Nurse.

[25] Nhung LH, Dien TM, Lan NP (2021). Use of Project ECHO Telementoring model in continuing medical education for pediatricians in Vietnam: preliminary results. Health Serv Insights.

[26] Tosi LL, Rajah EN, Stewart MH (2020). The rare bone disease teleecho program: leveraging telehealth to improve rare bone disease care. Curr Osteoporos Rep.

[27] Furlan AD, Zhao J, Voth J (2019). Evaluation of an innovative tele-education intervention in chronic pain management for primary care clinicians practicing in underserved areas. J Telemed Telecare.

[28] Arora S, Kalishman S, Thornton K (2010). Expanding access to hepatitis C virus treatment-Extension for Community Healthcare Outcomes (ECHO) project: disruptive innovation in specialty care. Hepatology.

[29] Tantillo M, Starr T, Kreipe R (2020). The recruitment and acceptability of a project ECHO^®^ eating disorders clinic: a pilot study of telementoring for primary medical and behavioral health care practitioners. Eat Disord.

[30] Queensland Government. Accessibility/Remoteness Index of Australia. 2019. https://www.qgso.qld.gov.au/about-statistics/statistical-standards-classifications/accessibility-remoteness-index-australia. Accessed 16 Mar 2023.

[31] The University of Sydney. ARIA Lookup Tool. 2022. https://www.pocog.org.au/aria/default.aspx. Accessed 16 Mar 2023

[32] Graneheim UH, Lundman B (2004). Qualitative content analysis in nursing research: concepts, procedures and measures to achieve trustworthiness. Nurse Educ Today.

[33] Bessell E, Kim JS, Chiem L (2022). Effectiveness of Project ECHO programs in improving clinician knowledge and confidence in managing complex psychiatric patients: a waitlist-controlled study. Acad Psychiatry.

[34] Newcomb D, Nixon P, Moss P, Kapoor V (2022). Supporting GPs in the management of children and young people with ADHD through Project ECHO^®^: results from a self-efficacy survey. Int J Integr Care.

[35] Ehrlinger J, Johnson K, Banner M (2008). Why the unskilled are unaware: further explorations of (absent) self-insight among the incompetent. Organ Behav Hum Decis Process.

[36] Dunning D, Heath C, Suls JM (2004). Flawed self-assessment implications for health, education, and the workplace. Psychological Science in The Public Interest.

[37] King R, Taylor B, Talpur A (2021). Factors that optimise the impact of continuing professional development in nursing: a rapid evidence review. Nurse Educ Today.

[38] Cervero RM, Gaines JK (2015). The impact of CME on physician performance and patient health outcomes: an updated synthesis of systematic reviews. J Contin Educ Health Prof.

[39] Reeves S, Fletcher S, Barr H (2016). A BEME systematic review of the effects of interprofessional education: BEME Guide No. 39. Med Teach.

